# Correlation between biological and mechanical properties of extracellular matrix from colorectal peritoneal metastases in human tissues

**DOI:** 10.1038/s41598-023-38763-w

**Published:** 2023-07-27

**Authors:** Ewelina Lorenc, Luca Varinelli, Matteo Chighizola, Silvia Brich, Federica Pisati, Marcello Guaglio, Dario Baratti, Marcello Deraco, Manuela Gariboldi, Alessandro Podestà

**Affiliations:** 1 Dipartimento di Fisica “Aldo Pontremoli” and CIMaINa, Università degli Studi di Milano, via G. Celoria 16, 20133 Milan, Italy; 2 Department of Research, Fondazione IRCCS Istituto Nazionale dei Tumori, via G. Venezian 1, 20133 Milan, Italy; 3 Department of Pathology and Laboratory Medicine, Fondazione IRCCS Istituto Nazionale dei Tumori, via G. Venezian 1, 20133 Milan, Italy; 4 Histopathology Unit, Cogentech Ltd. Benefit Corporation with a Sole Shareholder, via Adamello 16, 20139 Milan, Italy; 5 Peritoneal Surface Malignancies Unit, Colon and Rectal Surgery, Fondazione IRCCS Istituto Nazionale dei Tumori, via G. Venezian 1, 20133 Milan, Italy

**Keywords:** Cancer microenvironment, Atomic force microscopy, Biological physics, Applications of AFM

## Abstract

Peritoneal metastases (PM) are common routes of dissemination for colorectal cancer (CRC) and remain a lethal disease with a poor prognosis. The properties of the extracellular matrix (ECM) are important in cancer development; studying their changes is crucial to understand CRC-PM development. We studied the elastic properties of ECMs derived from human samples of normal and neoplastic PM by atomic force microscopy (AFM); results were correlated with patient clinical data and expression of ECM components related to metastatic spread. We show that PM progression is accompanied by stiffening of the ECM, increased cancer associated fibroblasts (CAF) activity and increased deposition and crosslinking in neoplastic matrices; on the other hand, softer regions are also found in neoplastic ECMs on the same scales. Our results support the hypothesis that local changes in the normal ECM can create the ground for growth and spread from the tumour of invading metastatic cells. We have found correlations between the mechanical properties (relative stiffening between normal and neoplastic ECM) of the ECM and patients’ clinical data, like age, sex, presence of protein activating mutations in *BRAF* and *KRAS* genes and tumour grade. Our findings suggest that the mechanical phenotyping of PM-ECM has the potential to predict tumour development.

## Introduction

Peritoneal Metastasis (PM) affects about one out of every four patients with colorectal cancer (CRC)^1^. PM development is characterised by several steps where cancer cells disseminate from the primary tumour to the peritoneal cavity^2^, through a process also known as peritoneal metastatic cascade^2,3^. To colonise the peritoneum, the neoplastic cells must be able to infiltrate the extracellular matrix (ECM), starting from detachment from the primary tumour, attach to sub-mesothelial connective tissue and receive a favourable host response^2^.

The ECM is an essential, acellular element of the tissue microenvironment, which plays a crucial role in several processes in tissue homeostasis^4^. The ECM determines the three-dimensional (3D) structure of the tissue and provides mechanical and biochemical support, playing a major role in cell–cell and cell–matrix communication and cell migration ^4,5^. Moreover, in the last decades, the crucial role of the ECM in cancer progression has been clearly demonstrated^6–11^.

The extracellular microenvironment is composed of water, various fibrous proteins (i.e. collagens, elastins, laminins, fibronectins), proteoglycans, glycoproteins, and polysaccharides; the ECM of the specific tissue has unique composition and topology, which results in developing the biochemical and mechanical properties of each organ and tissue^1,4,5^.

The ECM can be considered a dynamic element of the tissue, as it undergoes several changes in its composition and rearrangements of its own components, through covalent and non-covalent modifications, which are associated with cells activity in tissue development, and also severe diseases and cancer progression^5,12^.

Both mechanical and biochemical changes in the ECM are regulated by growth factors, hormones, cytokines and metalloproteinase (MMP)^6^. The ECM elastic properties, together with the activity of specific biochemical factors, play a key role in tissue homeostasis, cell fate, cell adhesion, migration, cell cycle progression, differentiation and actin-related cytoskeletal reorganisation and contractility^6,12–14^. The matrix is a multi-scale biomechanical entity that shows complex mechanical characteristics such as viscoelasticity, mechanical plasticity, and non-linear elasticity^8,15^. Viscoelastic and also biochemical properties of ECM are mostly determined by collagens and their cross-linking degree^8,15^. AFM studies of ECMs confirmed viscoelastic properties of this tissue compound^16,17^. There is increasing evidence that cells may sense and react to the viscoelastic, rather than to the static mechanical properties of ECMs^15^.

During cancer progression, the ECM undergoes many structural and biochemical changes, such as an increase of collagen deposition, fibres cross-linking and also changes in gene expression^4–6,12–14^. Indeed, the stiffening of the ECM can be observed in pre-malignant and malignant tissues^6,12^, is associated with high malignancy/aggressiveness and worse prognosis^7,13,14,18^ and leads to enhanced treatment resistance in most of the tumours^6^.

Cancer associated fibroblasts (CAFs) can originate from different cell types, including resident fibroblasts and mesothelial cells, which undergo a mesothelial-to-mesenchymal transition (MMT)^1,19^ and are critical for the progression of the metastatic disease. CAFs oversee the production of ECM proteins such as collagen, fibronectin, and several others as well as proteases and other enzymes involved in post-transcriptional modification of ECM proteins^19–21^. CAF activation and collagen deposition, which lead to an overall increase of ECM elastic modulus (stiffening) are among the signs of cancer progression. Therefore, the detection of ECM stiffening at the cellular scale could allow us to spot the first signs of tumour development and monitor cancer progression from its beginning. Moreover, a better understanding of the ECM stiffening process and the associated cell-ECM interplay could help develop more efficient therapeutic strategies for the prevention or treatment of cancer.

Atomic force microscopy (AFM) is a powerful and versatile tool to study biological samples at the nano- and microscale, including the quantitative investigation of their morphological and mechanical properties at multiple length scales^22–29^. The mechanical properties of cells, ECMs and tissues can be characterized by AFM^17,25–27,30–35^ and could constitute a unique mechanical fingerprint of cancer progression^36^.

Our work started from the hypothesis that the AFM study of nanomechanical properties of cells, ECMs and tissues, when complemented with the analysis of the expression of specific ECM components and with clinical metadata, can provide an important contribution to understanding the mechanisms that lead to the development of the PM. We have therefore studied the changes in the mechanical properties of the peritoneal ECM in patients affected by CRC-PM. In particular, we have characterized the Young’s modulus of elasticity of ECM specimens through indentation measurements performed by AFM^25^. The results of the nanomechanical analysis have been correlated with CAF presence and collagen organisation in the ECM samples, to obtain information on the physicochemical differences between normal and neoplastic ECMs, and with patient metadata to try to identify mechanical markers related of specific physiopathological state.

## Results

### Changes in the nanomechanical properties of the ECM

**Figure 1:**
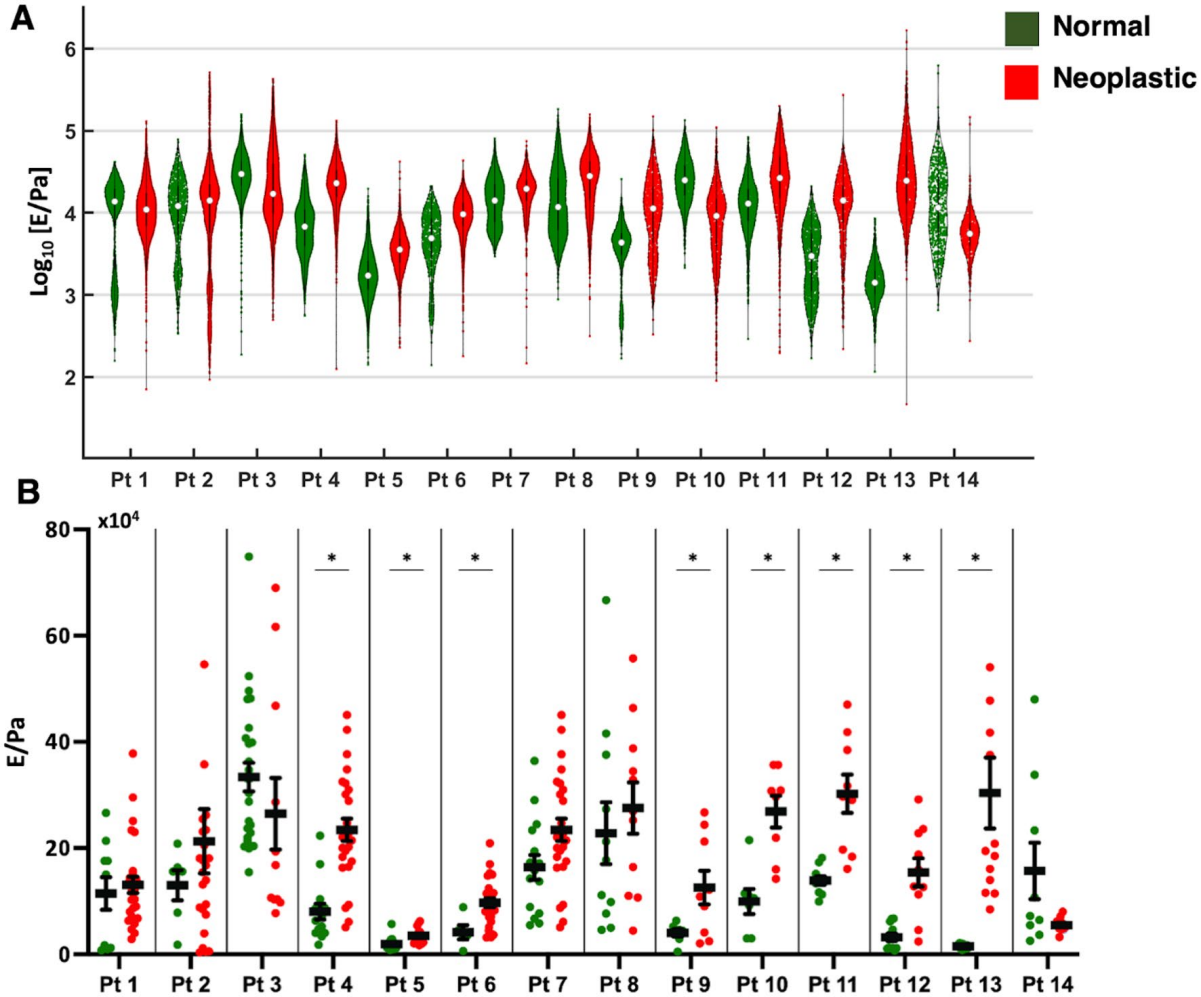
YM distributions for the normal (green) and neoplastic (red) conditions from peritoneal ECMs for the 14 patients considered in the study. (**A**) Violin plots obtained by pooling all YM values from all FCs acquired in all regions of interest (ROIs) for a specific condition. The median value is represented by a white dot and black thick lines represent upper and lower quartiles. (**B**) Plots showing the distribution of median YM values measured from all force volumes (FVs) collected in different ROIs for each specific condition (green and black dots). Black bars represent the mean of the median values and the corresponding standard deviation of the mean, respectively (see “Statistics” section). The asterisk indicates statistical significance of the difference (p < 0.05).

The heterogeneity of the ECM samples studied can be appreciated from the violin plots shown in Fig. 1A. In several cases, the YM distribution appears as clearly multimodal. It turns out that often the YM value of the highest-order mode is similar (i.e., the distribution shows significant overlap) to the YM value of a leading mode in the distribution of the neoplastic sample (see for example patients 1,2,3,6,7,8).

Figure 1B shows the distribution of the median YM values measured at different locations of the ECM samples for the tested conditions and patients. In some cases, stiffening is statistically significant, in others it is not, although an increase in the median YM value is often observed. Statistically significant stiffening (i.e., increase of the YM value from the normal to the neoplastic condition) of the CRC-PM derived ECM was observed for eight patients (4–6 and 9–13), who were also among the oldest: 82, 66 and 71 years, respectively, for patients 4–6 and 63, 67, for patients 10–13. However, the stiffening process was also present in patients 7, 8 and 9, who are significantly younger (47, 43 and 45 years old) (Fig. 1B).

For both normal and neoplastic tissues, the distribution of YM values is rather broad. Within the same tissue, we observed very wide patient-to-patient variability. For example, for the normal tissue, we observed a difference factor of ~ 17 between the YM value of patients 5 and 3; for the neoplastic condition, we observed a difference factor of ~ 8 between the YM value of patients 8 and 5. These results highlight, among other aspects, the importance of identifying internal references within the same patient; in our case, this is represented by the normal ECM collected several centimeters away from the cancer lesion.

### Correlation of the mechanical fingerprint with αSMA overexpression and collagen fibers presence

**Figure 2:**
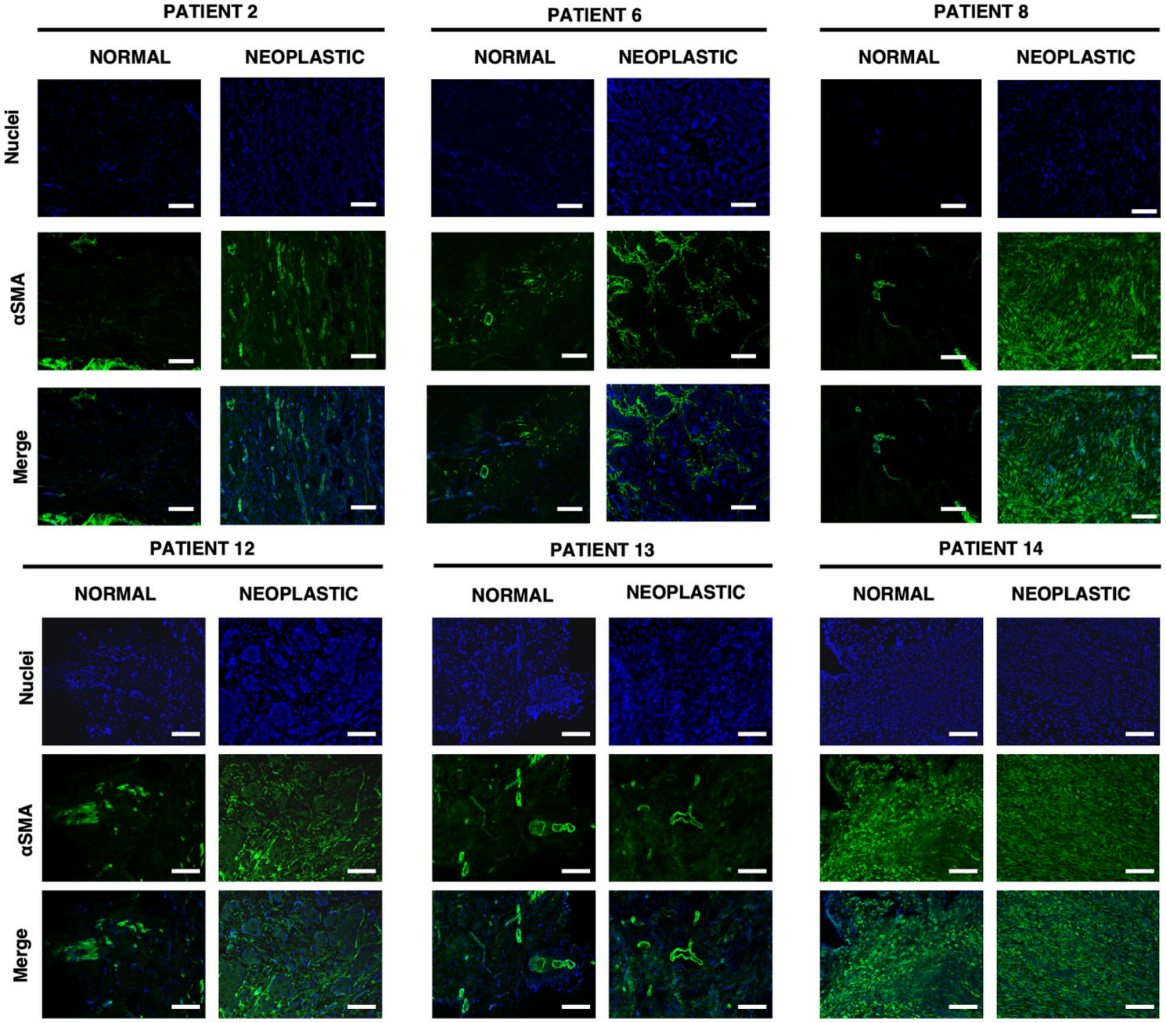
Images of tissue samples from patients 2, 6, 8 and 12–14, for visualisation of cell nuclei (DAPI staining) and αSMA, magnification 10x; scale bar length is 50 µm.

**Figure 3:**
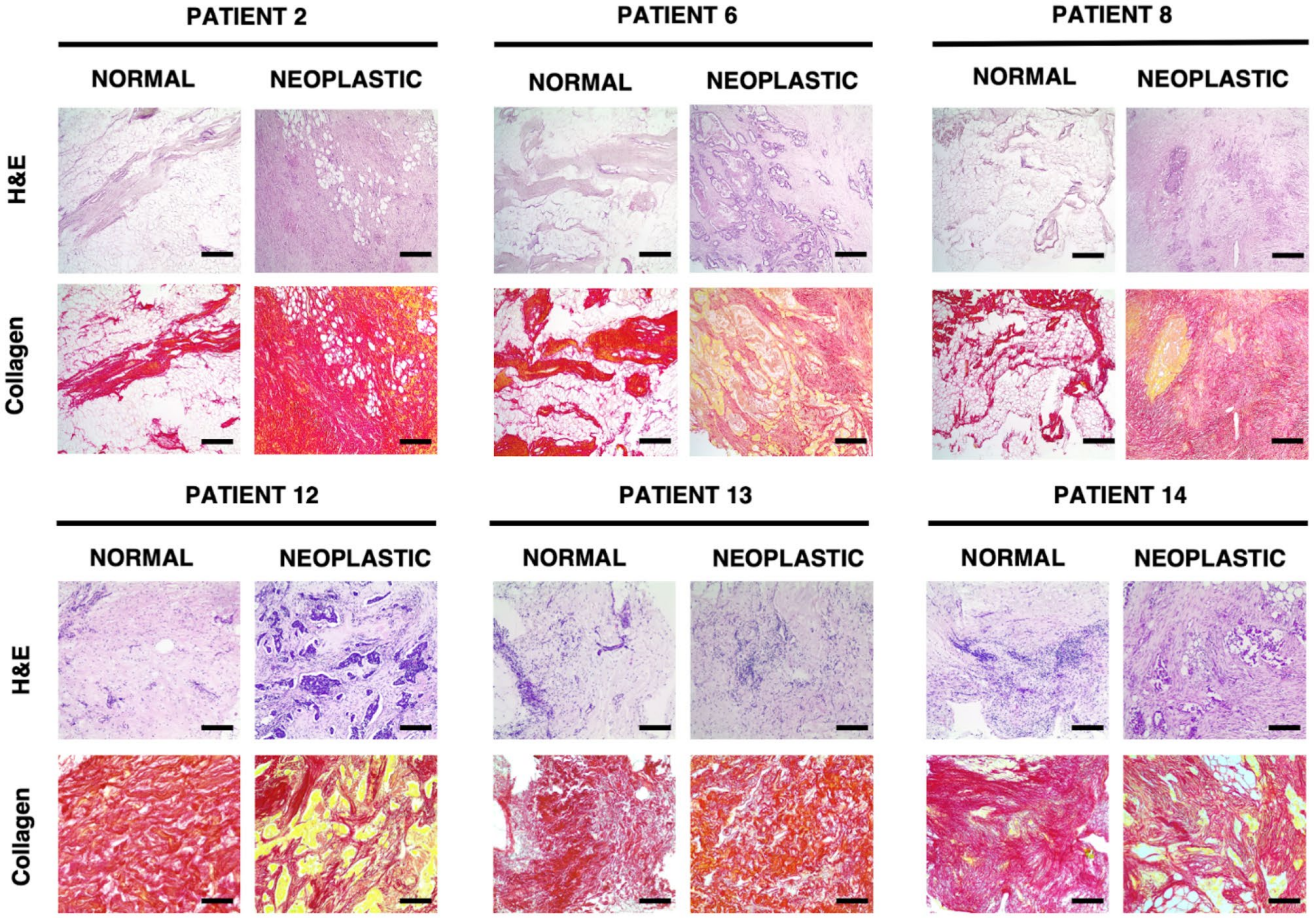
Images of tissue samples from patients 2, 6, 8 and 12–14, for visualization of collagen and control staining (Picrosirius Red and haematoxylin and eosin—H&E—staining, respectively), magnification ×4; scale bar length is 100 µm.

To better understand the biological events that sustain the mechanical properties of the ECM, we selected six different cases (patients) and analysed CAF activity related to collagen deposition and orientation by αSMA, and Picrosirius Red staining (Figs. 2 and 3). Patients whose ECMs exhibit different mechanical properties (no mechanical differences, moderate and significant stiffening between normal and neoplastic dECM) were selected.

Five of the six patients showed low expression of αSMA in normal-derived samples, although stromal regions showed high expression of localised αSMA (Fig. 2). Interestingly, we also observed high expression of αSMA around blood vessels (see patients 6, 8 and 13), a sign of the process of neo-angiogenesis, a well-known hallmark of cancer and metastatic spread. Neoplastic-derived samples showed higher expression of αSMA in patients 2, 6, 8, 12; while no clear differences in CAF activity were observed in patients 13 and 14. Patient 13 showed low expression of αSMA in both normal and neoplastic tissues, while patient 14 showed high expression of αSMA in both tissues. For three out of six patients, clear differences in αSMA expression (see Table 1) correlated with ECM stiffening, while patient 14 showed no differences in αSMA and no difference was observed in the measured YM of the two conditions. As can be seen in the case of normal-derived samples, αSMA expression is present, suggesting that neoplastic modifications of the environment are already occurring in a perilesional area (i.e., a region of the tissue that is healthy but close to the tumour mass). In patients 2, 8 and 12 (normal) stiffness distribution was bimodal (Fig. 1A) and a similar distribution was observed for αSMA expression.

**Table 1:** Mechano-biological relevant cancer-related modifications.

Patients	αSMA	Collagen	Mechanics	Correlation
2	+	++	+	*✔*
6	+	++	++	*✔*
8	++	++	*–*	
12	++	+	++	*✔*
13	*−*	*−*	++	
14	*−*	*−*	*–*	*✔*

+ : observable differences, ++ marked differences (statistically significant in mechanics), −: no observable differences.

We then evaluated the orientation of collagen fibers by Picrosirius Red staining (Fig. 3). The results showed that normal samples had higher deposition of collagen fibers in stromal and blood vessel areas (Fig. 3), while neoplastic-derived samples were characterized by an irregular and porous orientation of collagen fibers, with a corrugated-like morphology pattern (Fig. 3, see patients 2, 6, 8, 12–14). Overall, collagen results correlated with αSMA expression (see Table 1), demonstrating the active role of CAF in collagen production and deposition during metastatic spread (Figs. 2 and 3). Again, we observed that some normal-derived samples exhibited a neoplastic-like collagen pattern, also in line with αSMA expression, particularly in patient 14.

### Correlation of the mechanical fingerprint with patients’ clinical data

To better understand how mechanical response and ECM modifications are related to PM, we looked for correlations between the observed biophysical properties and clinical data of the patients.

**Figure 4:**
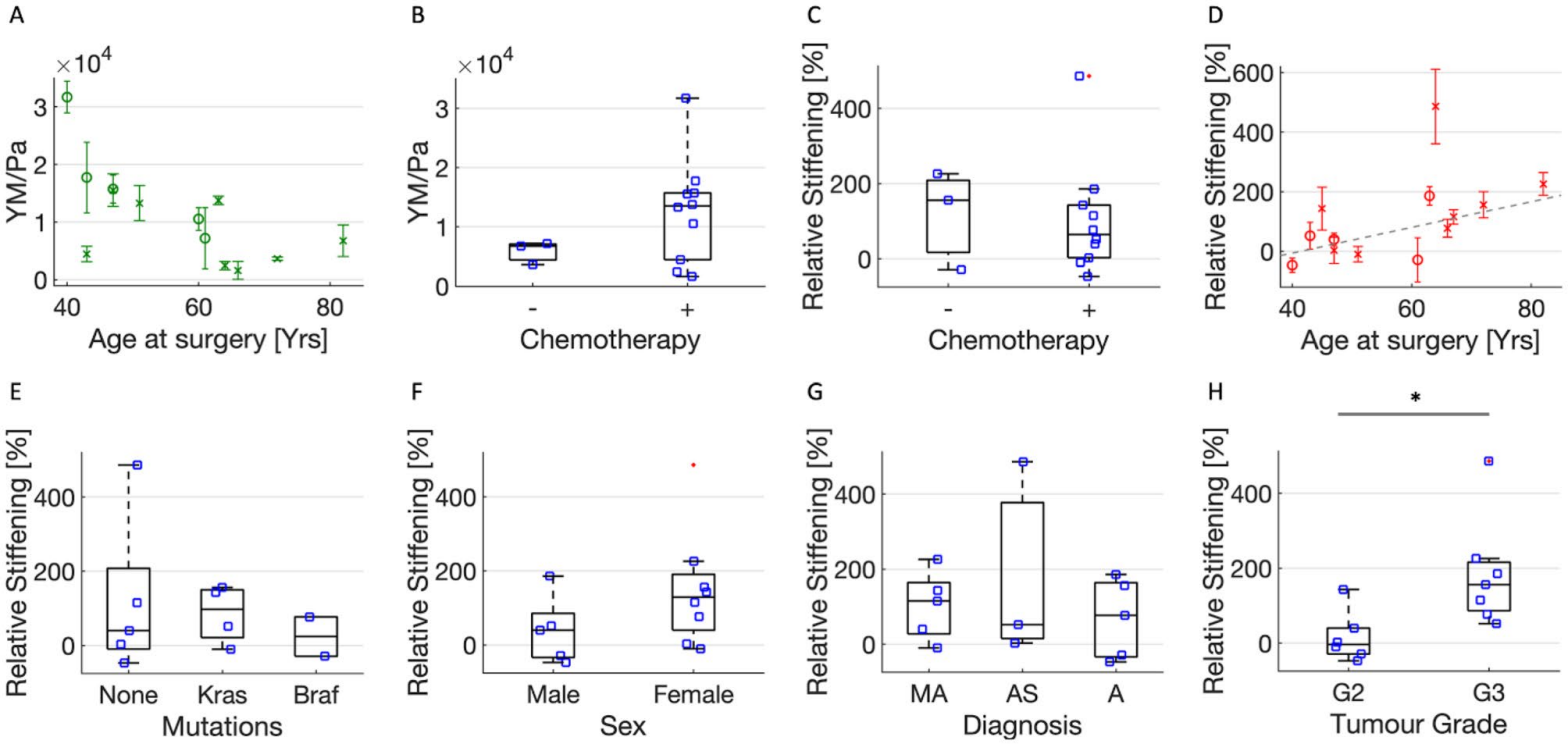
(**A**) YM values (mean median value ± std of the mean) of healthy ECMs of the 13 patients affected by adenocarcinoma versus their age (circles and crosses represent men and females, respectively). (**B**) YM values as in (**A**) for patients who did not (−) and did (+) undergo chemotherapy. (**C–H**) Relative stiffening of the neoplastic ECMs from the same patients, versus: (**C**) chemotherapic treatment; (**D**) the patients’ age; (**E**) the presence of protein activating mutations in *KRAS* and *BRAF* genes; (**F**) the patients’ sex; (**G**) histology—*MA* mucinous adenocarcinoma, *AS* adenocarcinoma of the sigma, *A* adenocarcinoma, *H* tumour grade.

We first tested whether the Young’s modulus of the normal ECM was correlated with the age of the patients involved, since age-related stiffening has been reported at both the cellular and ECM/tissue level^37–41^. The results are shown in Fig. 4A. It is possible to observe a clear trend toward softening of the normal ECM as the age of patients increases (patient 13, with a colorectal neuroendocrine carcinoma, not included in Fig. 4A, showed a decrease in line with the general trend).

To investigate whether the softening could be due to the treatments undergone by the patients, we checked for correlation with chemotherapy (Fig. 4B), but we found no significant evidence of its impact on the elastic properties of the ECM, despite a broader distribution of YM values for patients treated with chemotherapy. To avoid previous treatment-related biases, we calculated the relative stiffening of neoplastic versus normal ECMs within each single patient, as both tissues underwent the same treatments. The relative stiffening was calculated as the difference between the YM values of the neoplastic and normal ECM, normalized to the YM value of the normal ECM. Figure 4C confirms that chemotherapy is not correlated to stiffening of the ECM.

Figure 4D shows that there is a trend toward increasing relative stiffening with the age of the patients. Note that patient 13, with a colorectal neuroendocrine carcinoma, showed the strongest increase in stiffness (up to 1200%, and four times larger than the second highest relative stiffening). The very high stiffening observed in patient 13, compared with the other patients, can be a sign of different mechanical modifications between tumour types. However, since neuroendocrine tumours are extremely rare, our observations are not statistically significant and a comparison with the PM group is not possible; patient 13 was therefore excluded from analysis reported in Fig. 4.

We tested whether stiffening is related to the presence of mutations in the *KRAS* and *BRAF* genes that constitutively activate the corresponding proteins (Fig. 4E). The presence/absence of these mutations is important for diagnosis (e.g., tumors with mutations in *BRAF* have a worse prognosis) and for the choice of the type of treatment to be administered to the patient, since drugs with selective action are available for the presence/absence of specific mutations. These mutations are very frequent in metastatic CRC and are routinely tested for therapeutic treatment selection. We observed a difference (although not significant) between the relative stiffening of ECMs carrying protein activating *KRAS* and *BRAF* mutations, which appeared to be greater in *KRAS* mutated cases. The non-mutated cases spanned a wider range of relative stiffening, encompassing that of the mutated cases.

In addition, we decided to look for correlations between relative stiffening and the sex of patients, since it is known that CRC is more frequent in males than females, although the differences in mortality appear to be insignificant^42^. Figure 4E shows that females have more pronounced stiffening, although not significant. There are no data on this issue, so it might be interesting to test for sex-related variations in the mechanical properties of other tissues, as in the case of cardiovascular disease, which is known to affect males more than females^43^.

We also tested whether there are differences in mechanical properties in relation to the histopathological classification of the tumour (Table 2). Figure 4G shows that there are no evident differences in the relative increase of the YM of the ECM of the different histologies.

**Table 2:** Characteristics of the patients from whom the tissue samples were obtained. All samples were at stage IV.

Patient	Age^a^	Mutations at CRC-related genes^b^	Sex	Diagnosis	Grade	Location	Chemotherapy
CRC-PM 1^c^	51	*KRAS* G12D	Female	Mucinous Adenocarcinoma	G2	Colon	Yes
CRC-PM 2	47	NONE	Female	Adenocarcinoma	G2	Sigma	Yes
CRC-PM 3	40	NONE	Male	Adenocarcinoma	G2	Colon	Yes
CRC-PM 4	82	ND	Female	Mucinous Adenocarcinoma	G3	Right colon	No
CRC-PM 5^c^	66	*BRAF* V600E	Female	Adenocarcinoma	G3	Right colon	Yes
CRC-PM 6	71	*KRAS* G12S	Female	Adenocarcinoma	G3	Colon	No
CRC-PM 7	47	*NONE*	Male	Mucinous Adenocarcinoma	G2	Rectum	Yes
CRC-PM 8	41	*KRAS* G12S	Male	Mucinous adenocarcinoma	G3	Sigma	Yes
CRC-PM 9^c^	43	*KRAS* G12S	Female	Mucinous Adenocarcinoma	G2	Right colon	Yes
CRC-PM 10^c^	60	ND	Male	Adenocarcinoma	G3	Colon	Yes
CRC-PM 11^c^	63	NONE	Female	Mucinous Adenocarcinoma	G3	Colon	Yes
CRC-PM 12	65	NONE	Female	Adenocarcinoma	G3	Sigma	Yes
CRC-PM 13	82	#	Female	Colorectal neuroendocrine carcinoma	#	#	#
CRC-PM 14	61	*BRAF* V600E	Male	Adenocarcinoma	G2	Colon	No

^a^Age at surgery.

^b^*KRAS* and *BRAF* genes were analysed. Protein-activating aminoacidic change is shown.

^c^See Varinelli et al. 2022^46^.

Finally, we analysed the differences in the tumour grade of the samples analysed. Tumour grade describes a tumour in terms of abnormalities of tumour cells compared with normal cells. A low grade indicates a slower growing tumour than a high grade. All patients were diagnosed with grade 2 and grade 3; the results of the correlation between tumour grade and relative stiffening are shown in Fig. 4H. It can be observed that the grade 3 group (G3) shows significantly greater stiffening than the grade 2 group (G2).

## Discussion

Changes in the nanomechanical properties of tissues are one of the hallmarks of tumour progression^4,44,45^ By understanding the processes behind them, we could exploit this knowledge to implement cancer diagnosis and other diseases based on nano- and microscale mechanical phenotyping. Here, we focused on the investigation of the mechanical modifications in decellularized ECM derived from CRC-PM.

Based on AFM studies on ECM samples from 14 patients, we observed a general trend of ECM stiffening during the development of the neoplastic lesion (Fig. 1). Our results agree with previously published data on ECM of other cancer types^10,46,47^.

The measured multimodal, not simply broad, distributions of YM values, suggest that the ECM possesses significant structural, compositional, and therefore also mechanical heterogeneity at the AFM measurement scale (10–100 µm). Moreover, the partial overlap of YM values distributions of different conditions (normal and neoplastic), revealed the complexity of disease progression during the metastatic process, which is characterised by high spatial heterogeneity at the cellular and supracellular scale. The seed and soil theory suggests that ECM undergoes changes, including mechanical ones, to prepare a microenvironment suitable for neoplastic cell proliferation^48^. The presence of stiffer regions in normal samples, comparable to those typical of the neoplastic cases, suggests that local changes that prepare the ground for metastatic invasion in the normal tissue occur far from the existing lesion, likely caused by the release of factors that can ultimately alter the mechanical properties of the ECM^48^. The common practice for cancer studies is to obtain non-tumoural sample 10–15 cm away from the tumour^31^. Our results on the mechanical properties of the ECM show that the practice of considering the tissue located 10–15 cm far from the cancer lesion as non-tumoural could be uncorrect. The tissues from which samples were obtained were collected during CRS-HIPEC (Cytoreductive surgery—hyperthermic intraperitoneal chemotherapy). AFM-analysis was performed therefore on tissues that were already in advanced metastatic state, which were useful for characterising the ECM of normal and tumor peritoneal areas. Candidate cohorts for screening based on AFM mechanics should probably include cases collected during diagnostic laparoscopy for the diagnosis of PM, when the disease is still localised to small areas and when the perilesional area is probably less affected by metastatic changes. One or more biopsies around the metastatic lesion will allow determination of the extent of disease and subsequent treatment decision.

**Figure 5:**
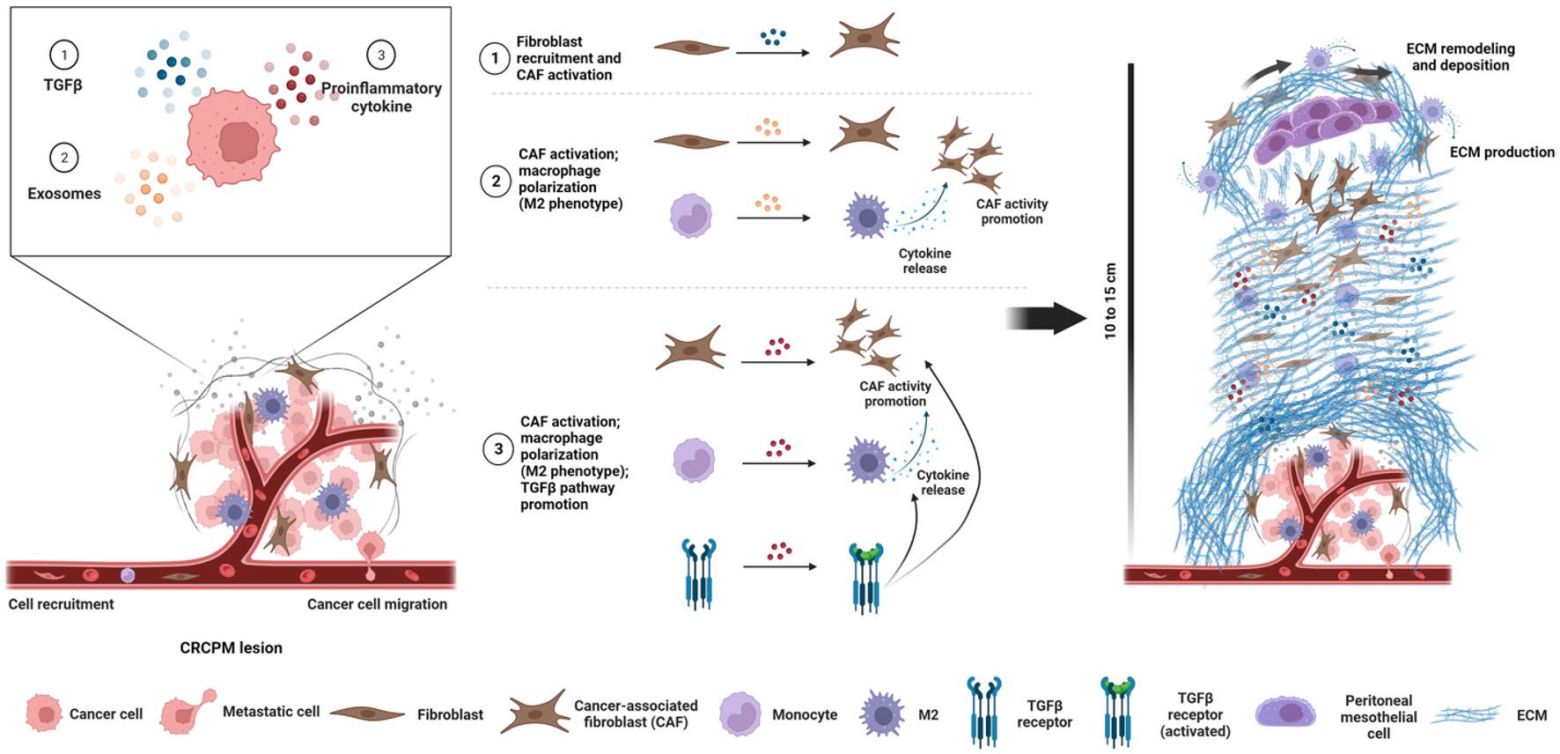
The figure summarises the main molecular and physical events that contribute to peritoneal metastatic niche development and ECM remodelling. Cancer cells exert their effect on the peritoneal metastasis microenvironment through three main modes: (1) the release of specific growth factors, such as TGFβ, leading to the recruitment of resident fibroblasts and their activation into CAF. (2) The production of exosomes that trigger the microenvironment and mediate the activation of CAFs and the polarisation of macrophages into M2 phenotype. (3) The production of pro-inflammatory cytokines, which contribute to enhancing the activity of CAFs and M2 macrophages by promoting the TGFβ pathway in a paracrine manner. Together, these events contribute to the production and/or deposition of naïve ECM and the concomitant increase in ECM stiffness, both around the site of injury and at distant sites in the peritoneal cavity, which may be a sign of disease progression.

Staining for αSMA, a CAF-specific protein expressed in fibroblasts, which is a sign of cancer progression and a typical marker of desmoplasia^19^, detected changes in the surrounding microenvironment that typically lead to the development of specific metastatic niches. Since we also observed areas with high expression of αSMA in normal samples, it is likely that these may have undergone modification induced by specific pro-metastatic factors released by nearby PM metastatic cells. These results confirmed that the formation of pre-metastatic niches already occurs in normal derived tissue as we also observed areas of high αSMA expression in normal samples (Fig. 5).

Higher expression of αSMA was also observed in areas rich in blood vessels (patients 6, 8 and 13). During the metastatic process, CAFs can distribute through blood vessels to develop the so-called perivascular metastatic niches, which can sustain the activation of stromal cells in normal tissue through TGF-β and secretion of pro-inflammatory and pleiotropic interleukins and cytokines, which also contribute to initiate modifications in the ECM, including stiffening. The combination of these events generates a microenvironment more suitable to support metastatic spread, in particular angiogenesis^19,21,48^. Expression of αSMA in vascularized areas indicates early steps of metastatic invasion into normal tissues (Fig. 5).

Expression of αSMA was higher than normal tissue in four of the six patients and CAF activity correlated with increased collagen deposition; nevertheless, two patients (8 and 13) exhibited uncorrelated results between staining and mechanical differences of normal and neoplastic ECM. Based on these results it appears that some of the tissue that was considered normal already had cancerous characteristics.

Another step to better understand the differences between normal and neoplastic ECM was to visualize collagen I and III fibers, as their overexpression and remodelling are strictly related to cancer progression^4–6,26,45^. Our results showed the typical expression and organization of collagen fibers already observed in previous studies on different types of ECM^10,34,49^. The organization of collagen fibers was strictly correlated with the expression of αSMA; expression of this protein confirmed collagen crosslinking in neoplastic ECM but also to a smaller extent in normal ECM. Tumours with high desmoplasia (fiber crosslinking) are considered more aggressive and with a worse prognosis^6^. In the neoplastic samples, increased crosslinking and restructuring of collagen fibrils in the ECM, and matrix stiffening produce an extracellular environment conducive to tumour invasion and growth. Changes in the vascularized and stiffer perilesional area could be a feed-forward loop to spread neoplastic ECM characteristics. Myofibroblasts are known for their ECM remodelling, which involves de novo deposition of specific receptors involved in mechanosignaling by the ECM, contributing to both normal and pathologic tissue remodelling^50^.

Correlation of these results with patients’ clinical data suggests a clear trend for tissue softening in older patients. This result is somewhat unexpected, considered that age-related stiffening at both cells and ECM/tissue levels has been reported^37–41^; damaging and inflammation of the tissue due to an extended inflammatory condition related to the presence of the tumour^32^ could explain our observation. We exclude that the softening can be directly related to chemotherapy treatments, since we did not find any evident correlations in our patients (Fig. 4B,C).

We then focused the analysis on the relative stiffening of neoplastic versus normal ECMs in each individual patient. The analysis of the mechanical properties according to the presence of mutations in *KRAS* and *BRAF* genes that determine constitutive activation of the corresponding proteins and across tumour grade showed that patients with mutations in *KRAS* gene had a slightly higher relative stiffening, while a stronger relative increase is related to different tumour grade (G3 > G2). Since the presence of protein activating mutations in *KRAS* and *BRAF* genes is very common in PM and tumour grade is a parameter that characterizes tumour cell behaviour, they are probably associated with specific mechanical characteristics. These data are still preliminary and will be investigated with further experiments on a larger cohort of cases. We believe that such correlations would help to advance the development of biomechanical tests to complement standard clinical diagnostic techniques.

Understanding how modifications of the mechanical properties of the ECM influence the metastatic invasion may also have the potential in developing active tissue treatments that can impact on cell migration; ECM is already being used as a scaffold for cell culture to better understand the cell-microenvironment interaction mechanisms^26,46,51–55^.

In conclusion, we demonstrated that in CRC-PM, ECM stiffening correlates with collagen deposition and remodeling, CAF activity, age of surgery, and tumor grade. Mechanical analysis with spatial resolution of human-derived samples revealed significant spatial heterogeneity in the elastic properties of normal and neoplastic ECM. The results, together with the high expression of αSMA, revealed that signs of pre-metastatic niche formation are already present in normal tissue, and correlation of the mechanical data with patient metadata showed interesting connections between relative stiffening and the characteristics of the tumor itself, particularly with the age and tumor grade of the patients. Our results suggest that nano- and microscale characterization of tissue mechanical properties may suggest the presence of metastasis and aid in diagnostic procedures.

Eventually, a more detailed chemico-mechanical investigation, considering the complete viscoelastic properties of the tissues and correlating them to the relative abundances of individual molecular components that characterize the ECM, would help to create a future database for elaborated diagnostic approaches based on mechanical quantitative study to complement standard clinical techniques.

## Materials and methods

### Sample preparation

ECMs were obtained from the peritoneal tissue of 14 patients diagnosed with CRC-PM (more detailed information is in Table 2).

The samples were collected during surgical resection at the Peritoneal Malignaces Unit of Fondazione IRCCS Istituto Nazionale Tumori di Milano as described in Varinelli et al*.*^46^ The study was approved by the Institutional Review Board of Fondazione IRCCS Istituto Nazionale Tumori di Milano (134/13; I249/19) and was carried out following the Declaration of Helsinki, 2009. All experiments were performed in accordance with relevant named guidelines and regulations. Written informed consent was obtained from all participants.

Briefly, non-tumoural tissues were collected 10 cm away from tumour, according to standard clinical procedures^31^. Neoplastic-derived and normal-derived 3D decellularized extracellular matrix (3D-dECM) specimens were obtained as described in Genovese et al.^56^ 3D-dECM were embedded in OCT and then frozen in a liquid nitrogen bath of isopropanol. Frozen samples from 2–3 patients at the same time were cut into slices of 100-200 µm thickness and immobilized on polarized glass slides (Thermofisher, Walthan, USA). Cryosections were stored at -20 °C and used for the AFM measurements immediately after preparation; the measurements of all prepared samples used to last up to two weeks, during which the samples to be studied in a specific session were took out of the freezer, thawed, and rinsed, as described below. In cases when longer storage was needed (more than 1 month), cryosections were kept in a freezer at -80 °C.

### AFM nanoindentation measurements

The nanomechanical measurements were performed at room temperature (approximately 25 °C), using a Bioscope Catalyst AFM (Bruker) mounted on top of an inverted microscope optical microscope (Olympus X71). To isolate the AFM from ground and acoustic noise, the microscope was placed on an active antivibration base (DVIA-T45, Daeil Systems) inside an acoustic enclosure (Schaefer, Italy).

Before AFM measurements, a cryosection was kept at room temperature for 15 min to melt OCT. Later, the cryosection was placed in a tube with cold PBS to wash away OCT. Presence of OCT could affect measurement by causing strong adhesion between tip and OCT. Additionally, measurements performed on ECM covered with OCT would prevent proper mechanical contact between tip and sample. Two washes were performed, 5–10 min each^57^.

AFM measurements of cryosections were performed in a PBS droplet confined by a circle of hydrophobic ink. The measurements were performed in PBS also to avoid tip-sample adhesion and ECM denaturation.

**Figure 6:**
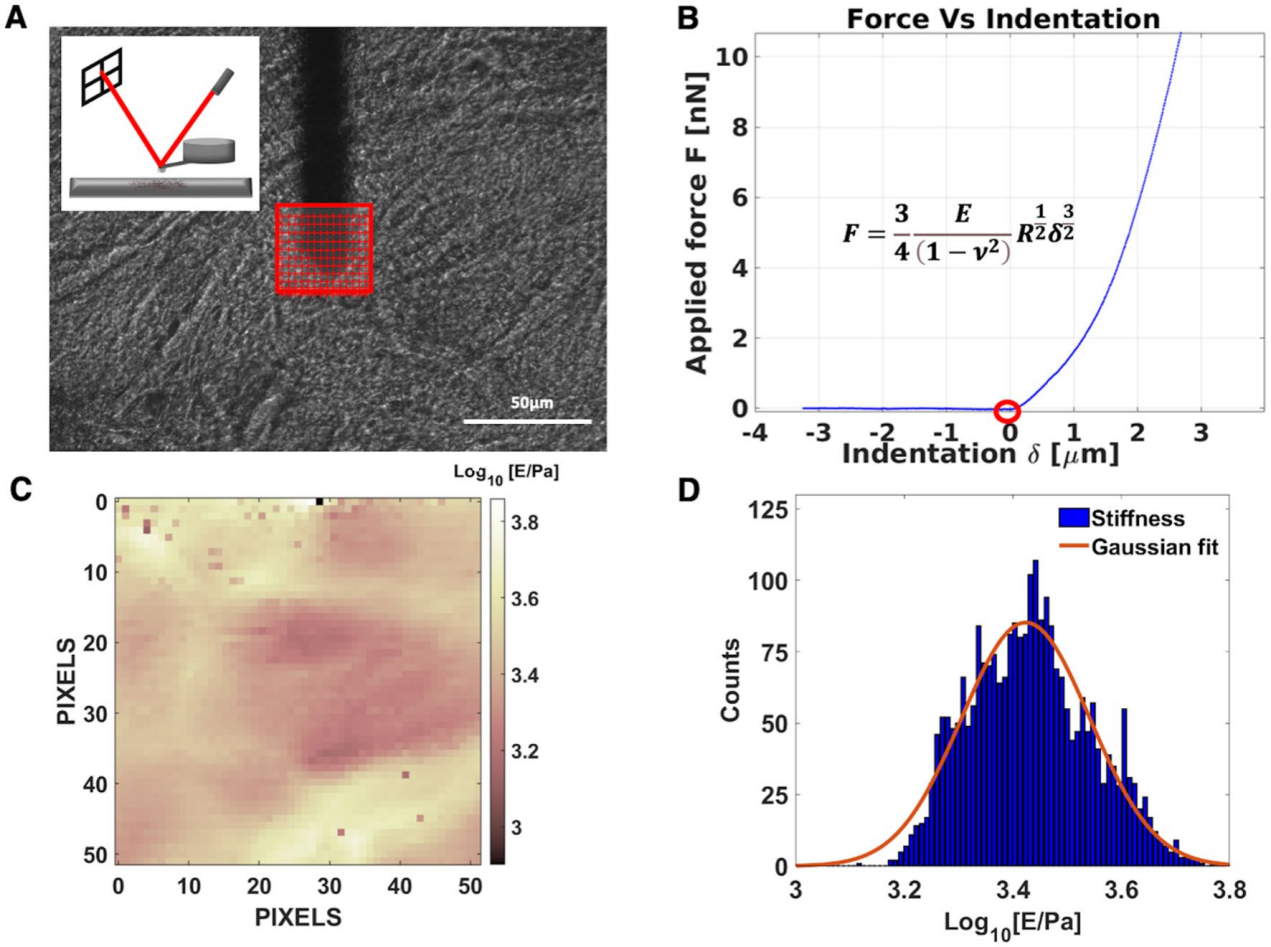
Schematic representation of the nanomechanical measurement. (**A**) Optical image of an ECM (normal peritoneum-derived from patient 8), with the AFM cantilever and the selected region of interest for the indentation experiment. Slices with thickness between 100 and 200 μm are semi-transparent, which allows to select regions for the analysis that look sufficiently uniform and smooth. The red grid represents the locations where force curves (FCs) are acquired (scale bar length—50 µm). In the inset, the experimental setup for indentation measurements and the optical beam deflection system are shown. (**B**) Typical rescaled approaching force vs indentation curve. The red circle highlights the contact point. Only the portion of the curve characterized by positive indentation is considered for the Hertzian fit (Eq. (1) and also shown in the inset). (**C**) The map of Young’s modulus values extracted by the FCs acquired in the region of interest shown in (**A**). (**D**) Histogram representing the distribution of YM values represented in the mechanical map in (**C**). Under the hypothesis of a log-normal distribution, a Gaussian fit in semi-log scale allows to identify the median YM value, as the centre of the Gaussian curve.

AFM-based nanomechanical measurements were performed according to standard procedures, based on the acquisition of indentation curves, as described in Refs^25,58^. The measurement process is described schematically in Fig. 6. We have used custom colloidal probes with spherical tips made of borosilicate glass beads with diameter (twice the radius R) in the range 18–25 µm, produced from Nanosensors TL-FM tipless cantilevers and calibrated as described in Ref.^59^. These large tips allow to effectively average the mechanical response of the structurally complex ECM. The spring constants of the AFM probes (typically 5–6 N/m, necessary to achieve indentations of several μm with a large tip) were calibrated using the thermal noise method^60,61^. The deflection sensitivity of the optical beam deflection apparatus (in units of nm/V) was calculated as the inverse of the slope of the force vs. distance curves (simply force curves, FCs) collected on a stiff substrate (the glass slide holding the sample)^58^ or using the contactless SNAP procedure^62^.

The samples were studied by collecting a set of FCs, also called force volumes (FVs), in different regions of interest (ROIs). Each FV typically covered an area between 50 µm × 50 µm to 125 µm × 125 µm and consisted of 100–225 FCs. The separation between adjacent FCs was chosen to be greater than the typical contact radius at maximum indentation, to reduce correlations between neighbouring FCs. For each patient’s condition (normal or neoplastic), several FVs were collected on different, macroscopically separated locations on each sample, with 2–3 samples (cryosections) per condition (normal vs neoplastic) for each patient. In total, for each condition 2000–4000 FCs were collected. A FC typically contained 8192 points, with ramp length L = 15 μm, maximum load F_max_ = 800–1500 nN, ramp frequency f = 1 Hz. The maximum load was adjusted to obtain a maximum indentation of 4–6 μm in all samples. Raw data were rescaled to produce force vs indentation curves in units of nN and nm, respectively, as explained in detail in Refs^25,61^.

Acquired data were analysed using custom MATLAB routines using protocol previously described in Puricelli et al.^25^. The elastic properties of the ECMs were characterised through their Young’s modulus (YM) of elasticity, extracted by fitting the non-adhesive Hertz model^63,64^ to the 20%-80% indentation range of the FCs (details in Ref.^23,25^):

1 $$F =\frac{4}{3}\frac{E}{1-{\nu }^{2}}{R}^\frac{1}{2}{\delta }^\frac{3}{2}$$

which is reported to be accurate as long as the indentation δ is small compared to the radius R. As a matter of fact, finite element analyses showed that Eq. (1) is accurate for δ as large as R, when micrometre-sized spherical beads are used as indenters (data unpublished, see also bottom effect -corrected results published in Ref.^65^). In Eq. (1), ν is the Poisson’s coefficient, which is typically assumed to be equal to 0.5 for incompressible materials, and E is the YM. Based on force curve inspection, we concluded that adhesion between the tip and the samples is negligible, therefore the Hertz model is expected to be accurate.

Finite thickness correction^25,66–68^ was not applied since the thickness of the ECM slices (150–200 μm) is significantly larger than the expected contact radius at maximum indentation. The first 20% of the FCs is typically ignored, due to the contribution of superficial non- crosslinked fibers, surface roughness issues, etc^10^.

### Histochemistry (HC) and immunofluorescence (IF) analyses

Before HC and IF staining, formalin-fixed-paraffin-embedded (FFPE) blocks were prepared and cut as in Varinelli et al.^46^. FFPE sections were stained with haematoxylin and eosin (H&E) for visualised nuclei and stromal regions. For HC analysis, sections were stained with picrosirius red (ScyTek lab), to visualise collagen fibers, following the manufacturers’ instructions. Antigen retrieval IF analysis was carried out as in Varinelli et al.^46^. For IF analyses, FFPE sections were stained with primary alpha Smooth Muscle Actin (αSMA,1:400), FITC conjugated antibody (Merck, KGaA) and DAPI (Merck, KGaA), following the manufacturers’ instructions, to visualise respectively cancer associated fibroblasts (CAF) and nuclei. Images were acquired with a DM6000B microscope (Wetzlar, Germany Leica,) equipped with a 100 W mercury lamp, and analysed using Cytovision software (Leica). All the experiments were performed in triplicate.

### Presence of protein activating mutations in KRAS and BRAF genes

The presence of protein activating mutations in *KRAS* and *BRAF* genes was determined using the Ion AmpliSeqTM Cancer Hotspot Panel (Thermo Fisher) which allows examination of hotspot regions of 50 oncogenes and tumor suppressor genes commonly mutated in human cancers, with broad coverage of the *KRAS* and *BRAF* genes. DNA from FFPE sections of PM was extracted as in Varinelli et al.^46^ and used for mutational analysis. The libraries were prepared using the IonAmpliSeq Library kit 2.0 (Thermo Fisher); emulsion PCR and chip loading were performed on the IonChef System (Thermo Fisher) and run on the IonGeneStudio S5 Prime (Thermo Fisher) using Ion 520 Chip and Ion 510 & Ion 520 & Ion 530 kit-chef according to the manufacturer's instructions. Raw data processing, variant calling and annotation were done as in Meazza et al.^69^.

### Statistics

The distribution of YM values that is peculiar of each ECM in the different tested conditions (Fig. 1A), has been created by pooling together all the FCs. This is justified in part by the fact that curve to curve distance is of the order of the maximum contact radius, and the separation between FVs from the same slice is comparable to the separation between FV from different slices obtained from the same patient. To highlight the diversity of local mechanical conditions met in the samples, we have used violin plots to represent the YM distributions.

The representative YM values for a specific condition of a specific patient has been done by grouping the median YM values obtained from each FV collected in different ROIs and calculating their mean value and the corresponding standard deviation of the mean^32^, assuming that the median values should be normally distributed according to the central limit theorem^70^. The distributions of median values are shown in Fig. 1B. An experimental relative error of approximately 3%, evaluated using a Monte Carlo method^71^, taking into account the uncertainties in the calibration factors (10% for the spring constant, 5% for the deflection sensitivity) was added in quadrature to the standard deviation of the mean to estimate the final error associated with the mean median YM values.

The statistical significance of differences between tested conditions was assessed using a two-tailed t-test. In case of a p-value < 0.05, the difference was considered as statistically significant.

## Data Availability

The datasets generated and/or analysed during the current study are available from the corresponding authors on reasonable request.
